# Carcinoma of the Ampulla of Vater: Determinants of Long-term Survival in 94 Resected Patients

**DOI:** 10.1155/1998/60259

**Published:** 1998-08

**Authors:** Jürgen Klempnauer, Gerd Jürgen Ridder, Hansjörg Maschek, Rudolf Pichlmayr

**Affiliations:** 1 Clinic of Abdominal and Transplantation Surgery Hannover Medical School Hannover D-30623 Germany; 2 Institute of Pathology Hannover Medical School Hannover D-30623 Germany; 3 Chirurgische Universitätsklinik Knappschaftskrankenhaus Bochum-Langendreer Ruhr-Universität Bochum Bochum D-44892 Germany

## Abstract

This retrospective study details 94 patients after surgical resection of carcinoma of the ampulla of Vater to determine prognostic factors. The tumour was limited to the ampulla of Vater in 32%, invaded the duodenal wall in 34%, infiltrated 2cm or less into the pancreas in 22%, and invaded more than 2cm into the pancreas and/or other adjacent structures in 11%. Curative resection was accomplished in 97% of cases. After exclusion of perioperative deaths the 1-, 5- and 10-year survival rates were 79.6%, 38.2%, and 31.6%, respectively with a median survival of 3.68 years. 26 patients survived more than five and 15 patients more than ten years. In an univariate analysis advanced tumour size, poor tumour grading, lymph node metastases and advanced UICC stage significantly decreased survival. Comparison of short and long survivors confirmed tumour size, lymph node status and UICC stage as significant prognostic factors. In a multivariate analysis (Cox model), only tumour size was a statistically independent predictor of prognosis. The survival probability increased with each year a patient survived after resection. When a patient had already survived five years after resection, the probability to survive another five years was 83%. Careful clinicopathologic staging is important for the prognosis after resection.


*HPB Surgery*, 1998, Vol. 11, pp. 1-11
Reprints available directly from the publisher
Photocopying permitted by license only

© 1998 OPA (Overseas Publishers Association) N.V.
Published by license under
the Harwood Academic Publishers imprint,
part of The Gordon and Breach Publishing Group.
Printed in India.

### Carcinoma of the Ampulla of Vater: Determinants of Long-term Survival in 94 Resected Patients

JÜRGEN KLEMPNAUER [a,*], GERD JÜRGEN RIDDER [a], HANSJÖRG MASCHEK [b] and RUDOLF PICHLMAYR [a]

[a] *Clinic of Abdominal and Transplantation Surgery, Hannover Medical School, D-30623 Hannover, Germany;*
[b] *Institute of Pathology, Hannover Medical School, D-30623 Hannover, Germany*

*(Received 21 September 1996; In final form 3 October 1997)*

**This retrospective study details 94 patients after surgical resection of carcinoma of the ampulla of Vater to determine prognostic factors. The tumour was limited to the ampulla of Vater in 32%, invaded the duodenal wall in 34%, infiltrated 2 cm or less into the pancreas in 22%, and invaded more than 2 cm into the pancreas and/or other adjacent structures in 11%. Curative resection was accomplished in 97% of cases. After exclusion of perioperative deaths the 1-, 5- and 10-year survival rates were 79.6%, 38.2%, and 31.6%, respectively with a median survival of 3.68 years. 26 patients survived more than five and 15 patients more than ten years. In an univariate analysis advanced tumour size, poor tumour grading, lymph node metastases and advanced UICC stage significantly decreased survival. Comparison of short and long survivors confirmed tumour size, lymph node status and UICC stage as significant prognostic factors. In a multivariate analysis (Cox model), only tumour size was a statistically independent predictor of prognosis. The survival probability increased with each year a patient survived after resection. When a patient had already survived five years after resection, the probability to survive another five years was 83%. Careful clinicopathologic staging is important for the prognosis after resection.**

*Keywords:* Ampullary carcinoma, ampullary neoplasms pathology, ampullary neoplasms surgery, prognostic factors, long-term prognosis

### INTRODUCTION

The prognosis of patients with malignant tumours is influenced by factors which can be categorised into patient-, tumour-, and treatment-associated factors [1]. In the present article, an effort is made to evaluate the prognostic significance of various clinicopathologic factors after surgical resection of ampullary carcinoma. The results of such analyses form the basis of a rational and pragmatic surgical treatment. Identification of prognostic factors will help to identify patients who have the chance of long-term survival and others who are at risk for an early tumour recurrence. The results of these statistical methods are fundamentally influenced by the chosen test variables and the method of

*Correspondence to: Professor Dr. med. J. Klempnauer, Chirurgische Universitätsklinik, Knappschaftskrankenhaus Bochum-Langendreer, Ruhr-Universität Bochum, D-44892 Bochum, Germany.

analysis. The majority of patients die within the first five years after resection and little is known about late survival of patients after surgery for carcinoma of the ampulla of Vater. In addition, it has been suggested that 5 year survival rates might underestimate the possibilities of later recurrence and death, making the real benefits of surgery doubtful. Thus, it appeared of interest to undertake a specific study of patients who underwent resection of ampullary cancer more than five and ten years ago to determine "who" are the long-term survivors after a resection of ampullary carcinoma. Within such a cohort of long-term surviving cancer patients one can identify those factors that are associated with either the absence or presence of long-term survival. A subtle analysis of the patient's individual situation contributes to an improved prognostic evaluation and grouping.

### PATIENTS, MATERIALS AND METHODS

### Patients Selection

At Hannover Medical School a total of 94 consecutive patients underwent resection of carcinoma of the ampulla of Vater between January 1971 and December 1995.

### Evaluation of Clinicopathological Data

Pathologic and operative notes were carefully reviewed to exclude any patient with a tumour arising from the duodenum, the intrapancreatic distal bile duct, the exocrine pancreatic tissue or the endocrine pancreas. In all 94 patients the resected specimens have been kept and were reanalysed for tumour grading. Tumour node metastasis (pTNM) staging was performed according to the staging system jointly developed by the American Joint Committee on Cancer (AJCC) and the International Union Against Cancer (UICC) [2]. The residual tumour (R) classification was used to define the absence or presence of residual tumour after resection. R1 represents microscopic, R2 macroscopic, and R0 no residual tumour. Tumour differentiation was determined using the UICC grading system, evaluating H and E stained sections of at least five tissue blocks per tumour sample. In our patients adjuvant chemotherapy was only applied in four patients, but without a standardised regimen of adjuvant or multimodal treatment.

### Data Collection

Data concerning patient, clinical and pathologic tumour characteristics, treatment modalities and their results were collected from medical records. Follow-up data were obtained by patient interview, letters, hospital charts, and personal contact with the attending physician. For survivors the attending physician was asked to fill out a standardized questionaire. Complete information about the survival status could be obtained for all patients. Follow-up data were available as from 1 April 1996. Operative mortality was calculated as death before hospital discharge.

### Statistical Analysis

Statistical analysis was performed using the chi-square ($\chi^2$) test with the probability level set to $p \leq 0.05$. Patient survival was calculated by the method of Kaplan and Meier [3]. The prognostic significance of each clinicopathological variable was calculated after exclusion of hospital mortality. The relationship between each of the variables and survival was assessed by the log rank test (Mantel–Cox) [4]. A value of $p \leq 0.05$ was considered to be statistically significant. A multivariate analysis was performed using Cox's proportional hazards regression [5]. Only the variables that were statistically significant by univariate analysis were included in a multivariate analysis to establish a hierarchy among the various prognostic factors. The stability of the model was certified by using a likelihood ratio step-forward and step-backward fitting procedure. The level of significance was taken from the last step of the regression analysis. A value of $p \leq 0.05$ was considered to be statistically significant.

Histopathological slides and resected specimens were reviewed to confirm the diagnosis and to study the following pathological features with potential prognostic influence: size and grading of the tumour, nodal status and margin, tumor localisation, distant metastases, vascular invasion, microscopic involvement of perineural spaces, and lymphatic and blood vessels. Other determinants analysed included age, sex, type of resection, radicality of operation, clinical symptoms (jaundice, diabetes mellitus, upper abdominal pain, back pain, *etc.*), intra- and postoperative blood transfusions, postoperative complications, and extended vascular and organ resection.

### RESULTS

### Patient Characteristics

There were 56 males (60%) and 38 females (40%) aged 34 to 82 years (median 63.1±11.4 years).

### Clinical Presentation

The presenting clinical symptoms were painless jaundice (60%), upper abdominal pain (37%), weight loss (35%), weakness (13%), loss of appetite (11%), and back pain (5%). Acute and chronic pancreatitis were noted in 6% and 4%, resp. Eight patients had a previous history of malignancy. The diagnosis was established by ultrasonography, computed tomography, endoscopy, and endoscopic retrograde cholangiography. Biopsies were not regularly taken.

### Surgical Procedures

Tumour removal was accomplished by partial duodenopancreatectomy in 91 cases. In 3 of these patients pylorus preserving Whipple's procedure was applied. Pancreatic reconstruction comprised pancreatojejunostomy in 84% and pancreogastrostomy in 16%. Three patients underwent total pancreatectomy. Intra- and/or postoperative blood transfusions were given in 84% of patients.

### Operative Mortality

Inhospital mortality was 9.6% (9 of 94) in the entire time period. In the last decade mortality amounted to 5.6%. Causes of death were septic multiorgan failure in all but one patient who died from massive bleeding. The underlying problem was insufficiency of the pancreatic anastomosis in 4 and of the biliodigestive anastomosis in one patient. Relaparotomy because of postoperative complications was necessary in 26%.

### pTNM Staging and Tumour Grading

The tumour was limited to the ampulla of Vater in 32% (pT1), invaded the duodenal wall in 34% (pT2), infiltrated 2 cm or less into the pancreas in 22% (pT3), and invaded more than 2 cm into the pancreas and/or other adjacent structures in 11% (pT4). Regional lymph node involvement was found in 38%. There was a direct correlation between tumour size and lymph node status.

Distant metastases were present only in one patient. Despite multiple small liver metastases a Whipple's operation was performed. Residual adenoma was found in a total of 8 of 94 (8.5%) of resected carcinomas. In 21 patients since 1991 the number of resected lymph nodes in the resected specimens was determined and amounted to a mean of 12.5±1. In nodal positive patients an average of 39±8% of the removed lymph nodes were affected.

According to the UICC the one patient with a tumour *in situ* was classified as stage 0. Twenty-six percent of patients were grouped into stage II, 30% in stage III, and 12% in stage IV.

In all cases an adenocarcinoma arose from the mucosa of the ampulla of Vater. Tumour grading was possible in all but six cases. Tumour was well (G1) in 40%, moderate (G2) in 49%, poorly differentiated (G3) in 10%, and undifferentiated (G4) in 1%.

### Residual Tumour Stage

Curative R0 resection was possible in all but three patients (97%). Microscopic tumour was left behind (R1 resection) at the pancreatic resection margin in two patients. Hepatic metastases were not resected in one patient (R2 resection).

### Long-term Survival and Tumour Recurrence

Overall survival including hospital deaths was 34.5% at 5 and 28.6% at 10 years. After exclusion of hospital mortality the respective figures were 38.2% at 5 and 31.6% at 10 years. The median survival time after exclusion of hospital deaths was 3.68±0.88 years. 26 patients actually survived for more than 5 and 15 for more than 10 years. Table I lists the clinicopathological factors of the 15 patients surviving more than ten years after curative resection of ampullary cancer in chronological sequence. The majority of these (8/15) is still alive with no evidence of disease. To our knowledge none of the remaining 7 patients died from tumour recurrence.

Table II lists a comparison of clinicopathological factors in patients who died prior to the median survival time or who survived beyond this time. Significant differences were noted for tumour size and UICC stage. Lymph node metastases were not significant ($p = 0.0628$), neither were age, gender, partial *vs.* total pancreatectomy, blood transfusions, and postoperative requirement for a relaparotomy.

Figure 1 shows the development of the five year survival probability for patients who have already survived various time intervals after resection of ampullary carcinoma. The chance to survive another 5 years increased with each year after resection. When a patient had already survived five years after resection the chance to survive another five year period was 83%.

### Uni- and Multivariate Analysis

Table III shows the clinicopathological parameters investigated in the univariate survival

TABLE I Clinicopathological factors in patients surviving ten years or longer after curative resection of ampullary carcinoma in chronological sequence

| Operation date | Age | Gender | pTNM | UICC-stage | Grading | Survival (years) | Outcome |
|---|---|---|---|---|---|---|---|
| 11/72 | 35.8 | f | T1 N0 M0 | I | GX | 16.6 | died from breast cancer |
| 4/76 | 66.2 | f | T2 N0 M0 | II | G1 | 19.9 | alive, NED |
| 2/77 | 56.0 | m | T3 N0 M0 | II | G3 | 17.4 | dead |
| 2/77 | 61.4 | m | T2 N0 M0 | II | GX | 11.4 | dead |
| 10/78 | 67.6 | m | T2 N0 M0 | II | G1 | 17.3 | alive, NED |
| 8/80 | 50.7 | f | T1 N0 M0 | I | G1 | 15.5 | alive, NED |
| 9/81 | 73.7 | m | T1 N0 M0 | I | G1 | 12.8 | dead, NED |
| 9/81 | 58.4 | f | T2 N1 M0 | III | G2 | 14.5 | dead, NED |
| 11/81 | 69.6 | f | T1 N0 M0 | I | G1 | 13.4 | dead, NED |
| 9/83 | 72.4 | f | T2 N1 M0 | III | G1 | 10.2 | dead, NED |
| 11/83 | 60.9 | m | T3 N1 M0 | III | G2 | 12.3 | alive, NED |
| 4/84 | 51.3 | m | T2 N1 M0 | III | G1 | 11.9 | alive, NED |
| 8/84 | 40.1 | m | T1 N0 M0 | I | G1 | 11.5 | alive, NED |
| 10/84 | 65.8 | f | T1 N0 M0 | I | GX | 11.3 | alive, NED |
| 11/85 | 40.9 | m | T2 N0 M0 | II | G3 | 10.3 | alive, NED |

NED: no evidence of disease.

TABLE II Comparison of clinicopathologic factors between patients who died prior to the median survival time of the entire group (3.68 years) and patients who survived beyond this time years

| Factor | Early deaths ($n$=39) | | Long survivors ($n$=36) | | $\chi^2$-test |
|---|---|---|---|---|---|
| Tumour size | | | | | 0.0107 |
| pTis | 1 | (3) | 0 | (0) | |
| pT1 | 8 | (21) | 13 | (36) | |
| pT2 | 8 | (21) | 16 | (44) | |
| pT3 | 15 | (38) | 6 | (17) | |
| pT4 | 7 | (18) | 1 | (3) | |
| Lymph node metastasis | | | | | 0.0628 |
| pN0 | 20 | (51) | 26 | (72) | |
| pN1 | 19 | (49) | 10 | (28) | |
| Distant metastasis | | | | | * |
| M0 | 38 | (97) | 36 | (100) | |
| pM1 | 1 | (3) | 0 | (0) | |
| UICC stage | | | | | 0.0436 |
| Stage 0 | 1 | (3) | 0 | (0) | |
| Stage I | 7 | (18) | 11 | (31) | |
| Stage II | 9 | (23) | 15 | (42) | |
| Stage III | 14 | (36) | 9 | (25) | |
| Stage IV | 8 | (21) | 1 | (3) | |
| Residual tumour stage | | | | | * |
| R0 | 37 | (95) | 36 | (100) | |
| R1 | 1 | (3) | 0 | (0) | |
| R2 | 1 | (3) | 0 | (0) | |
| Tumour grading | | | | | 0.3581 |
| well differentiated (G1) | 12 | (32) | 16 | () | |
| moderately differentiated (G2) | 20 | (54) | 12 | () | |
| poorly differentiated (G3) | 4 | (11) | 4 | () | |
| undifferentiated (G4) | 1 | (3) | 0 | (0) | |
| Preoperative jaundice | | | | | 0.2054 |
| Present | 28 | (76) | 21 | (62) | |
| Absent | 9 | (24) | 13 | (38) | |

Values in parentheses are percentages. Analysis excluded hospital mortality. Only complete data were taken into account. *statistical analysis not performed, because of too few patients.

analysis. Tumour size, lymph node metastases, UICC stage and grading had a significant impact on long term survival. The Kaplan–Meier survival plots for the different tumour sizes are depicted in Figure 2. No statistical significance for survival was attached to age, gender, type of resection, blood transfusions, postoperative complications requiring relaparotomy and the presence of preoperative jaundice. Distant metastases and residual tumour stage were not tested because of too few patients.

In the multivariate analysis only tumour size was of independent prognostic significance ($\chi^2 = 12.89$, $p = 0.0118$).

### DISCUSSION

The objective of this study was to identify determinants of long-term survival after resection of ampullary carcinoma. Our retrospective single centre analysis comprised 94 patients who all had tumours arising in the mucosa of the ampulla of Vater. In the literature, the histogenetic origin of periampullary tumours is not always clearly defined [6]. Ampullary, distal bile duct, duodenal, and pancreatic carcinoma have to be distinguished [7]. An accurate pathologic confirmation of the tumour type and anatomic origin is essential when reporting survivial rates

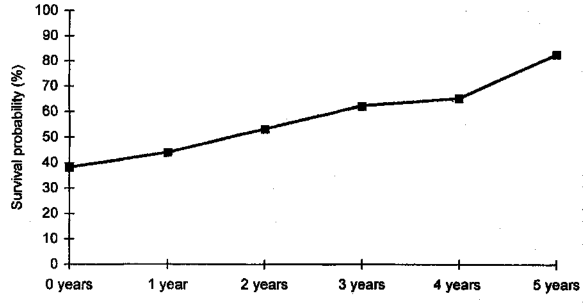

| years | 0 | 1 | 2 | 3 | 4 | 5 |
|---|---|---|---|---|---|---|
| patients at risk | 85 | 64 | 49 | 40 | 34 | 26 |

FIGURE 1 Development of the five year survival probability for patients who have already survived various time intervals after resection of carcinoma of the ampulla of Vater.

and factors for prognostic grouping for ampullary carcinoma [1]. There is a close relationship between adenoma and adenocarcinoma of the ampulla of Vater. In our series coexisting adenoma was found in 8.5%. Adenomas of the ampulla of Vater are not encountered so frequently as those in the colon and rectum, but an ampullary adenocarcinoma can arise from a pre-existing adenoma, in the same location [8, 9].

There has been a considerable debate whether there is a place for local excision of ampullary carcinomas [10–12]. Theoretically, local resection is only applicable for stage 0 and I tumours. Pre- and intraoperatively, however, it cannot be precisely determined whether the tumour is limited to the ampulla of Vater itself or whether

TABLE III Univariate survival analysis according to clinicopathological parameters

| Factor | *n* | 5 year survival (%) | 10 year survival (%) | *p* (log rank) |
|---|---|---|---|---|
| Tumour size | | | | 0.0057 |
| pTis | 1 | died after 2.5 yrs | | |
| pT1 | 25 | 56.0 | 43.6 | |
| pT2 | 29 | 50.6 | 45.5 | |
| pT3 | 21 | 14.3 | 9.5 | |
| pT4 | 9 | 22.2 | 0 | |
| Lymph node metastasis | | | | 0.0162 |
| pN0 | 53 | 44.9 | 36.8 | |
| pN1 | 32 | 27.2 | 22.6 | |
| Distant metastasis | | | | * |
| M0 | 84 | 38.7 | 32.0 | |
| pM1 | 1 | died after 0.4 yrs | | |
| UICC stage | | | | 0.0488 |
| Stage 0 | 1 | died after 2.5 yrs | | |
| Stage I | 22 | 58.1 | 49.8 | |
| Stage II | 26 | 40.8 | 31.7 | |
| Stage III | 26 | 29.7 | 23.8 | |
| Stage IV | 10 | 20.0 | 0 | |
| Residual tumour stage | | | | * |
| R0 | 83 | 39.1 | 32.4 | |
| R1 | 1 | died after 0.2 yrs | | |
| R2 | 1 | died after 0.4 yrs | | |
| Tumour grading | | | | < 0.0001 |
| well differentiated (G1) | 29 | 46.9 | 43.0 | |
| moderately differentiated (G2) | 40 | 26.3 | 16.4 | |
| poorly differentiated (G3) | 9 | 51.9 | 38.9 | |
| undifferentiated (G4) | 1 | died after 0.4 yrs | | |
| Preoperative jaundice | | | | 0.4283 |
| Present | 53 | 35.5 | 27.9 | |
| Absent | 28 | 40.8 | 35.7 | |

Analysis excluded hospital mortality. Only complete data were taken into account.
*statistical analysis not performed, because of too few patients.

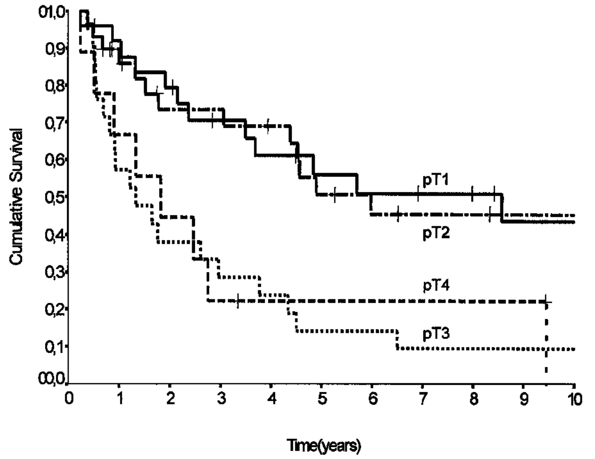

FIGURE 2 Kaplan-Meier survival according to tumour size (log rank test, $p = 0.0024$). Hospital mortality excluded. Tick mark indicates last follow-up.

it infiltrates adjacent structures. Mucosal spread or interstitial infiltration was frequently found even in cases with carcinoma at a relatively early stage [13]. Final tumour staging is only possible after definitive histopathological examination of the resected specimen. Thus, pancreatoduodenectomy should be the treatment of choice for all carcinomas of the ampulla of Vater [14–19]. During duodenopancreatectomy the pancreas is transsected at the left border of the mesentericoportal vein. In our view a systematic and radical lymphadenectomy is obligatory. The pylorus-preserving modification of Whipple's operation has been advocated for resection of ampullary carcinomas [20, 21]. Advantages of pylorus preservation are a more physiologic alimentary reconstruction and the fact that there is no reflux of bile into the stomach. It has to be assured, however, that the extent of lymphadenectomy is not impaired [21]. Lymph node involvement around the stomach is very rare in carcinoma of the ampulla of Vater [21, 22].

There is a considerable mortality and morbidity of local resection, which may exceed that of Whipple's procedure [10, 11]. Hospital mortality rate after pancreatic resection decreased in the present series, from more than 20 per cent in the earlier period to about 5 per cent in the later period. The operative mortality rate could be dramatically reduced in all centres dealing with pancreatic surgery, and some authors have reported a large number of pancreatic resections without deaths [19, 23, 24]. We have excluded the postoperative mortality from the uni- and multivariate factorial analyses. The inclusion of postoperative deaths makes the direct comparison of results dubious, because otherwise hospital mortality would be a primary prognostic factor.

In order to characterise the prognostic factors of ampullary cancer we have used three different approaches namely uni- and multivariate analysis, comparison between short and long-term survivors and evaluation of patients who actually survived 10 years after resection. Uni- and multivariate analyses identify various prognostic factors after resectional therapy of ampullary carcinoma and provide more reliable information than that obtained only through clinical experience or simple statistical analysis. Regression analyses clarify the extent to which each factor has statistical independence. Because of the fact, that the majority of patients dies within the first few years after surgical tumour resection, it is important to analyse determinants of long-term survival. We have thus compared the clinicopathological factors of patients who died prior and after the median survival time of the entire group. It was of interest to evaluate patients who actually survived for prolonged periods of time, since one and 5 year survival rates underestimate the possibilities of later recurrence and death.

The only independent prognostic factor derived from multivariate Cox proportional hazard regression was tumour size. Tumour size was not measured as actual diameter in centimetres. The qualitative definition of the UICC was employed which considers the depth of local invasion. Tumours limited to the ampulla of Vater had an excellent prognosis. Even when the tumour directly invaded the duodenal wall, more than 40% of patients were still alive at 10 years after surgery. The prognosis was markedly impaired when the tumour had invaded the pancreas and/or other adjacent structures. Besides tumour size other factors of prognostic significance were lymph node status, grading, and the UICC stage. These factors, however, had no independent influence on prognosis when integrated in the regression analysis and proved to be significant only in the univariate approach.

With respect to the lymph node status the lack of significance in the multivariate analysis can be partially attributed to the fact, that patients with advanced tumour size also had a higher proportion of positive lymph nodes. In the univariate approach positive lymph nodes at the time of resection were clearly associated with an impaired prognosis. These findings are in accordance with data from the literature [25]. In all cases a systematic and radical lymphadenectomy was performed in our patients. From our data it cannot be delineated whether such a lymphadenectomy has a beneficial effect on prognosis. Removal of regional lymph nodes, however, is absolutely mandatory for an adequate tumour staging.

A high degree of tumour differentiation was also significantly correlated with a more favourable prognosis in the uni- but not the multivariate regression analysis. The prognostic relevance of tumour grading after resection of ampullary cancer is not surprising. It is also a feature of ductal pancreatic carcinoma and other gastrointestinal adenocarcinomas.

The UICC staging system has the deliberate objective to predict the individual patient's prognosis at the time of resection. Unfortunately, this staging system proved to be only of limited value after resection of ampullary carcinoma. From our retrospective analysis this is mainly related to an overemphasis in the UICC classification of the lymph node status over tumour size. In our opinion, however, tumour size is more important than lymph node status. In particular, stage II comprises both nodally negative pT2 and pT3 tumours. Our data show, that direct infiltration of ampullary tumours into the pancreas was associated with a dramatically impaired prognosis, whereas infiltration of the duodenum did not decrease the survival probability in comparison to pT1 tumours which are limited to the ampulla of Vater itself. Furthermore, the UICC places patients with positive lymph nodes generally in stage III and does not further distinguish between pT1, pT2 and pT3 tumours. In a smaller group of 36 patients Sperti *et al.*, identified tumour stage, lymph node involvement and tumour differentiation as prognostic factors but only in a univariate analysis [26].

It seems evident that distant metastases and non-curative resections also have a negative impact on prognosis. In our analyses, these two factors did not prove to be significant simply because of a too small number of patients involved. Diffuse small liver metastases were left behind during a Whipple's operation and the patient died after 0.4 years. Non-curative resection with infiltration of the pancreatic resection margin in two patients was also associated with very short survival of only a few months.

Our analysis also revealed factors which had no effect on prognosis after resection of ampullary cancer *e.g.*, age and sex, blood transfusions, surgical complications, and preoperative jaundice. Age should not be a contraindication to resection. Perioperative blood transfusion has been identified as a negative prognostic factor in a number of solid tumours [27–29]. Cameron *et al.*, noted that patients receiving more than 2 units of blood in the perioperative period after resection for pancreatic cancer had a significantly worse prognosis, with transfusion proving to be an independent prognostic factor [30].

In our opinion, however, the adverse influence of blood transfusion may well be the result of a correlation with other prognostic variables, since transfused patients are very often those who present with poorer performance status, with

TABLE IV Published results of resection in patients with carcinoma of the ampulla of Vater

| Author | Year of publication | Patient no. | Mortality rate | Survival |
|---|---|---|---|---|
| Wise *et al.* *[34] | 1976 | 2390 | 18.8% | 29.3% |
| Tarazi *et al.* [35] | 1986 | 105 | 7.8% | 37.2% |
| Yamaguchi and Enjoji† [9] | 1987 | 109 | 6% | 28% |
| Neoptolemos *et al.* [36] | 1988 | 23 | NA | 52.1% |
| Dawson and Connolly [37] | 1989 | 24 | 12.5% | 29% |
| Mori *et al.* [38] | 1990 | 24 | 8.3% | 50.2% |
| Monson *et al.* [39] | 1991 | 104 | 5.7% | 34% |
| Bakkevold and Kambestad [40] | 1993 | 30 | NA | 15% |
| Willett *et al.* [41] | 1993 | 41 | 4.9% | 55% |
| Matory *et al.* [42] | 1993 | 55 | 3.6% | 43.0% |
| Sperti *et al.* [26] | 1994 | 36 | 2.8% | 56% |
| Andersen *et al.* [43] | 1994 | 25 | NA | 34% |
| Nakao *et al.* [44] | 1994 | 26 | 0% | 52% |
| Roder *et al.* [25] | 1995 | 66 | 4.5% | 35% |
| Chan *et al.* [45] | 1995 | 29 | NA | 43% |
| Futakawa *et al.* [13] | 1996 | 60 | NA | NA |
| present study | 1996 | 94 | 9.6% | 38.2% |

NA = not available; *collected series; †multicenter study.

more advanced and rapidly spreading diseases and/or undergo more problematic surgery. Randomised prospective studies, encompassing a score of problems faced during surgery, and a well-defined policy of blood transfusion in the post-operative period will further highlight this topic. In our series adequate management of complications did not impair long-term prognosis. Our experience could not confirm that non-icteric ampullary carcinoma have a more favourable prognosis [31]. About 30% of patients with ampullary carcinomas are not icteric at time of diagnosis [31, 32].

The prognosis after resection of ampullary carcinoma is excellent especially when compared to ductal pancreatic cancer [33]. In this respect our study confirms the results reported from many other centers (Tab. IV). We would like to speculate that a considerable number of patients are actually cured from ampullary carcinoma by surgical resection. Our experience is that patients who have survived five years and more have a life expectancy which resembles that of a normal population. Other authors have pointed out that five-year survival offered no guarantee of cure. Trede *et al.* [23] noted that 5 of his 21 patients with 5 year survivial subsequently died of their cancer, and Monson *et al.*, reported that 8 of 20 patients surviving 5 years after resection died of tumour recurrence [39]. Characterisation of long-term survivors after surgery for cancer of the ampulla of Vater is particularly useful to better define the selection criteria for treatment. It is essential to define patients who have a high risk to die early after resection. This subgroup might benefit from adjuvant multimodal cancer treatment including chemo- and or radiotherapy. In patients with a good chance of long survival, surgical resection might suffice alone and additional chemo- and radiotherapy would be overtreatment.

### References

[1] Hermanek, P., Hutter, R. V. P. and Sobin, L. H. (1990). Prognostic grouping: the next step in tumor classification. *J. Cancer Res. Clin. Oncol.*, **116**, 513–516.

[2] Hermanek, P., Scheibe, O., Spiessl, B. and Wagner, G. (1992) TNM classification of malignant tumours. Fourth Edition, 2nd Revision, Berlin, Springer.

[3] Kaplan, E. L. and Meier, P. (1958). Nonparametric estimation from incomplete observations. *J. Am. Stat. Assoc.*, **53**, 457–481.

[4] Mantel, N. (1966). Evaluation of survival data and two new rank order statistics arising in its consideration. *Cancer Chemother Rep.*, **50**, 163–170.

[5] Cox, D. R. (1972). Regression models and life tables. *J. Roy. Stat. Soc.*, **34**, 187–200.

[6] Rumpf, K. D., Ostertag, H. and Pichlmayr, R. (1986). Das periampulläre Karzinom – Ein fragwürdiger Terminus. In: Berger, H. G. and Bittner, R. (Eds.) Das Pankreaskarzinom, Springer, Berlin, pp. 349–355.

[7] Michelassi, F., Erroi, F., Dawson, P. J., Pietrabissa, A., Noda, S., Handcock, M. and Block, G. E. (1989). Experience with 647 consecutive tumors of the duodenum, ampulla, head of the pancreas, and distal common bile duct. *Ann. Surg.*, **210**, 544–556.

[8] Stolte, M. and Pscherer, C. (1996). Adenoma-carcinoma sequence in the papilla of Vater. *Scand J. Gastroenterol.*, **31**, 376–382.

[9] Yamaguchi, K. and Enjoji, M. (1987). Carcinoma of the ampulla of Vater: A clinicopathologic study and pathologic staging of 109 cases of carcinoma and 5 cases of adenoma. *Cancer*, **59**, 506–515.

[10] Asbun, H. J., Rossi, R. L. and Munson, J. L. (1993). Local resection for ampullary tumors. Is there a place for it? *Arch Surg.*, **128**, 515–520.

[11] Gadzijev, E. and Pegan, V. (1992). Extended excision of the ampulla of Vater–a new operative technique for elderly patients. *Hepatogastroenterol*, **39**, 475–477.

[12] Jones, B. A., Langer, B., Taylor, B. R. and Girotti, M. (1985). Periampullary tumors: which ones should be resected? *Am. J. Surg.*, **149**, 46–52.

[13] Futakawa, N., Kimura, W., Wada, Y. and Muto, T. (1996) Clinicopathological characteristics and surgical procedures for carcinoma of the papilla of Vater. *Hepatogastroenterol.*, **43**, 260–267.

[14] Bakkevold, K. E., Arnesjø, B. and Kambestad, B. (1992). Carcinoma of the pancreas and papilla of Vater: Presenting signs, and diagnostics related to stage and tumour site. *Scand J. Gastroenterol.*, **27**, 317–325.

[15] Böttger, T., Zech, J., Weber, W., Sorger, K. and Junginger, T. (1989). Prognostisch relevante Faktoren beim Carcinom der Papilla Vateri. *Langenbecks Arch. Chir.*, **374**, 358–362.

[16] Crist, D. W. and Cameron, J. L. (1992). The current status of the whipple operation for periampullary carcinoma. *Adv. Surg.*, **25**, 21–49.

[17] Hayes, D. H., Bolton, J. S., Willis, G. W. and Bowen, J. C. (1987). Carcinoma of the ampulla of Vater. *Ann. Surg.*, **206**, 572–577.

[18] Shutze, W. P., Sack, J. and Aldrete, J. S. (1990). Long-term follow-up of 24 patients undergoing radical resection for ampullary carcinoma, 1953 to 1988. *Cancer*, **66**, 1717–1720.

[19] Trede, M. (1993). The surgical options. In: Trede M. and Carter D. C. (Eds.) Surgery of the Pancreas. Churchill Livingstone, Edinburgh, pp. 433–442.

[20] Grace, P. A., Pitt, H. A. and Longmire, W. P. (1986). Pancreatoduodenectomy with pylorus preservation for adenocarcinoma of the head of the pancreas. *Br. J. Surg.*, **73**, 647–650.

[21] Traverso, L. W. and Longmire, W. P. (1980). Preservation of the pylorus in pancreaticoduodenectomy: a follow-up evaluation, *Ann. Surg.*, **192**, 306–310.

[22] Kimura, W. and Ohtsubo, K. (1988). Incidence, sites of origin, and immunohistological and histochemical characteristics of a typical epithelium and minute carcinoma of the papilla of Vater. *Cancer*, **61**, 1394–1402.

[23] Trede, M., Schwall, G. and Saeger, H. D. (1990). Survival after pancreatoduodenectomy. 118 consecutive resections without an operative mortality. *Ann. Surg.*, **211**, 447–458.

[24] Cameron, J. L., Pitt, H. A., Yeo, C. J., Lillemoe, K. D., Kaufmann, H. S. and Coleman, J. (1993). One hundred and forty-five consecutive pancreaticoduodenectomies without mortality. *Ann. Surg.*, **217**, 430–438.

[25] Roder, J. D., Schneider, P. M., Stein, H. J. and Siewert, J. R. (1995). Number of lymph node metastases is significantly associated with survival in patients with radically resected carcinoma of the ampulla of Vater. *Br. J. Surg.*, **82**, 1693–1996.

[26] Sperti, C., Pasquali, C., Piccoli, A., Sernagiotto, C. and Pedrazzoli, S. (1994). Radical resection for ampullary carcinoma: Long-term results. *Br. J. Surg.*, **81**, 668–671.

[27] Barra, S., Barzan, L., Maione, A., Cadelano, A., Pin, M., Franceschi, S. and Comoretto, R. (1994). Blood transfusion and other prognostic variables in the survival of patients with cancer of the head and neck. *Laryngoscope*, **104**, 95–98.

[28] Tartter, P. I. (1992). The association of perioperative blood transfusion with colorectal cancer recurrence. *Ann. Surg.*, **206**, 633–638.

[29] Wu, H. and Little, A. (1988). Perioperative blood transfusions and cancer recurrence. *J. Clin. Oncol.*, **6**, 1348–1354.

[30] Cameron, J. L., Crist, D. W., Sitzmann, J. V., Hruban, R. H., Boitnott, J. K., Seidler, A. J. and Coleman, J. (1991). Factors influencing survival after pancreaticoduodenectomy for pancreatic cancer. *Am. J. Surg.*, **161**, 120–125.

[31] Yamaguchi, K., Enjoji, M. and Kitamura, K. (1990). Non-icteric ampullary carcinoma with a favorable prognosis. *Am. J. Gastroenterol.*, **85**, 994–999.

[32] Ridder, G. J. and Klempnauer, J. (1996). Presenting symptoms of cancer of the exocrine pancreas and the periampullary region: Established facts and new insights from an analysis of surgically treated patients. *Zentralbl Chir.*, **121**, 557–564.

[33] Klempnauer, J., Ridder, G. J. and Pichlmayr, R. (1995). Prognostic factors after resection of ampullary carcinoma: multivariate survival analysis in comparison with ductal cancer of the pancreatic head. *Br. J. Surg.*, **82**, 1686–1691.

[34] Wise, L., Pizzimbono, C. and Dehner, L. P. (1976). Periampullary cancer: a clinicopathologic study of sixty-two patients. *Am. J. Surg.*, **131**, 141–148.

[35] Tarazi, R. Y., Hermann, R. E., Vogt, D. P., Hoerr, S. O., Esselstyn, C. B.Jr., Cooperman, A. M., Steiger, E. and Grundfest, S. (1986). Results of surgical treatment of periampullary tumors, a thirty-five year experience. *Surgery*, **100**, 716–722.

[36] Neoptolemos, J. P., Talbot, I. C., Shaw, D. C. and Carr-Locke, D. L. (1988). Long-term survival after resection of ampullary carcinoma is associated independently with tumor grade and a new staging classification that assesses local invasiveness. *Cancer*, **61**, 1403–1407.

[37] Dawson, P. J. and Connolly, M. M. (1989). Influence of site of origin and mucin production on survival in ampullary carcinoma. *Ann. Surg.*, **210**, 544–556.

[38] Mori, K., Ikei, S., Yamane, T., Yamaguchi, Y., Katsumori, T., Shibata, Y. and Arai, T. (1990). Pathologic factors influencing survival of carcinoma of the ampulla of Vater. *Eur. J. Surg. Oncol.*, **16**, 183–188.

[39] Monson, J. R. T., Donohue, J. H., McEntee, G. P., McIlrath, D. C., Heerden, J. A. van, Shorter, R. G., Nagorney, D. M. and Ilstrup, D. M. (1991). Radical resection for carcinoma of the ampulla of Vater. *Arch. Surg.*, **126**, 353–357.

[40] Bakkevold, K. E. and Kambestad, B. (1993). Long-term survival following radical and palliative treatment of patients with carcinoma of the pancreas and papilla of Vater – the prognostic factors influencing the long-term results. A prospective multicentre study. *Eur. J. Surg. Oncol.*, **19**, 147–161.
[41] Willett, C. G., Warshaw, A. L., Convery, K. and Compton, C. C. (1993). Patterns of failure after pancreaticoduodenectomy for ampullary carcinoma. *Surg. Gynecol. Obstet.*, **176**, 33–38.
[42] Matory, Y. L., Gaynor, J. and Brennan, M. (1993). Carcinoma of the ampulla of Vater. *Surg. Gynecol. Obstet.*, **177**, 366–370.
[43] Andersen, H. B., Baden, H., Brahe, N. E. B. and Burcharth, F. (1994). Pancreaticoduodenectomy for periampullary adenocarcinoma. *J. Am. Coll. Surg.*, **179**, 545–552.
[44] Nakao, A., Harada, A., Nonami, T., Kishimoto, W., Takeda, S., Ito, K. and Takagi, H. (1994). Prognosis of cancer of the duodenal papilla of Vater in relation to clinicopathological tumor extension. *Hepatogastroenterol.*, **41**, 73–78.
[45] Chan, C., Herrera, M. F., Garza, L. de la, Quintanilla-Martinez, L., Vargas-Vorackova, F., Richaud-Patin, Y., Llorente, L., Uscanga, L., Robles-Diaz, G., Leon, E. and Campuzano, M. (1995). Clinical behavior and prognostic factors of periampullary adenocarcinoma. *Ann. Surg.*, **222**, 632–637.

### COMMENTARY

This retrospective review of one unit's experience with the treatment of carcinoma of the ampulla of Vater provides important information to guide us in our treatment strategies for this disease. The authors are in the unique position of having access to detailed pathologic records and specimens of all patients who underwent resectional surgery since 1971. Furthermore they were able to correlate the pathologic data with patient survival in all 94 patients consecutively treated in the one institution. The results were not surprising but give the following strong messages; Major pancreatic surgery performed in experienced units is associated with an acceptably low operative mortality consequently resectional surgery for attempted cure should be considered for all patients with this disease. Patients with ampullary carcinoma can be cured following surgical resection. Cure and long term survival is dependent on the tumour characteristics such as depth of local invasion, tumour grading and lymph node metastases.

Preoperative assessment of the tumour related prognostic factors may now be possible with advances in endoscopic and laparoscopic ultrasound techniques [1]. Recent studies have shown that endoscopic ultrasonography can quite accurately determine the depth of invasion of ampullary tumours and may provide information that can be used in determining the type of therapy to be recommended [2]. For instance if ultrasonography determines that the tumour is confined to the mucosa it may be appropriate to limit the surgery to local resection.

Preoperative laparoscopic assessment and laparoscopic ultrasonography has been shown to detect with high accuracy lymph node metastases and spread to the liver [3]. Survival of these patients is not prolonged by major resectional surgery consequently these minimal access techniques have been used in order to exclude patients who will not benefit from major surgery.

The results of the study by Klempnauer *et al.*, provides the pathologic and survival data on which assessment of ultrasonographic staging may be based.

### *References*

[1] John, T. G. and Garden, O. J. (1996). Laparoscopic ultrasound for diagnosis of pancreatic conditions. In *Endosurgery*, Eds., Toouli, J. Gossot, D. and Hunter, J. Churchill Livingstone London, pp. 583–598.
[2] Rosch, T., Braig, C., Gain *et al.* (1992). Staging of pancreatic and ampullary carcinoma by endoscopic ultrasonography. Comparison with conventional sonography computed tomography and angiography. *Gastroenterology*, **102**, 188–199.
[3] John, T. G., Greig, J. D., Carter, D. C. and Garden, O. J. (1995). Carcinoma of the pancreatic head and periampullary region: tumor staging with laparoscopy and laparoscopic ultrasonography. *Annals of Surgery*, **221**, 156–164.

Prof. J. Toouli
Head of Gastrointestinal Surgical Unit
Department of Surgery
Flinders Medical Centre
Bedford Park, Adelaide
South Australia
Australia

